# Warshaw Technique in Spleen-Preserving Pancreatectomy: A Case Series

**DOI:** 10.7759/cureus.79211

**Published:** 2025-02-18

**Authors:** Spiros Delis, Dimosthenis Chrysikos, Dimitris Liatsos, Despoina Sperdouli, Konstantinos Alifieris, Amir Shihada, Michail Saintanis, Nikos Kokoroskos, Andreas Palantzas, Konstantinos Liakopoulos, Theodore Troupis

**Affiliations:** 1 Department of General Surgery, Konstantopoulio General Hospital, Athens, GRC; 2 Department of Anatomy, National and Kapodistrian University of Athens School of Medicine, Athens, GRC

**Keywords:** pancreatic ca, spleen preservation, surgical treatment, total pancreatectomy, warshaw technique

## Abstract

We present three cases of spleen-preserving total pancreatectomy using the Warshaw technique for neoplasms of the pancreatic head. The first case involved a 5 cm mucinous pancreatic neoplasm invading the middle colic artery. The second case featured a patient with a BMI of 40 and adenocarcinoma of the pancreatic head. The third case involved a serous cystadenoma in the pancreatic head in a patient with a history of gastric surgery for a gastric ulcer, requiring surgical intervention due to weight loss and abdominal symptoms. These cases highlight the surgical nuances and benefits of the Warshaw technique, particularly its role in preserving splenic blood supply and reducing postoperative complications.

## Introduction

Pancreatic cancer incidence has been rising, accounting for 2% of all cancer cases and 5% of cancer-related deaths. Despite advancements in modern medicine, early diagnosis remains challenging, contributing to its extremely low five-year survival rate of 2-9% [1]. Treatment typically involves a combination of surgery, chemotherapy, radiotherapy, targeted therapy, and immunotherapy, with surgical resection playing the most significant role in prolonging survival [1,2]. The most common surgical procedures include total pancreatectomy, distal pancreatectomy with splenectomy, and pancreaticoduodenectomy [1]. These interventions are primarily performed in patients with resectable tumors (i.e., no extension to the superior mesenteric artery) and borderline resectable tumors (i.e., encirclement of less than 180 degrees of the superior mesenteric artery’s circumference) [2].

Despite its crucial role in treating pancreatic cancer, pancreatic surgery has historically been associated with high morbidity and mortality rates. While improvements in surgical techniques have reduced these risks to more acceptable levels, they remain a significant concern [3,4].

A major factor contributing to the complexity of pancreatic surgery is splenectomy, which is often performed following pancreatic resection [5]. The rationale for splenectomy is both oncological, to ensure adequate lymph node dissection, and technical, to facilitate the removal of tumors near the splenic hilum [5,6]. However, literature has not demonstrated a clear prognostic benefit or improved lymph node yield from splenectomy in pancreatic cancer cases. Moreover, splenectomy increases the risk of postoperative infections, including life-threatening post-splenectomy sepsis from encapsulated bacteria. Additionally, it can lead to gastric venous congestion and left-sided portal hypertension, often presenting with thrombocytosis [5,6].

To mitigate these complications, spleen-preserving surgery should be considered in patients with nonmalignant tumors and expanded as an alternative approach to reduce the risk of postoperative pancreatic fistula (POPF) [6,7]. Two primary techniques for spleen preservation have been described: the Warshaw and Kimura techniques [7].

The Warshaw technique, introduced by Andrew L. Warshaw in 1988, involves entering the lesser sac through the gastrocolic ligament, which is divided to allow stomach mobilization and full visualization of the pancreas, including its tail and the splenic hilum. Care is taken to preserve the short gastric and left gastroepiploic vessels, which supply collateral blood flow to the spleen. The avascular plane posterior to the pancreatic tail is dissected, allowing the pancreas to be mobilized into a “free-floating bridge.” The splenic artery and vein are then clamped, divided, and ligated at their origin, while the spleen itself remains untouched unless injury occurs. Before completing the procedure, the spleen’s color, size, and viability must be assessed [5,8].

In contrast, the Kimura technique preserves the native splenic vessels, posing a greater technical challenge for the surgeon [6,7].

Spleen-preserving total pancreatectomy presents unique challenges, particularly in cases involving neoplastic lesions or significant vascular involvement. The Warshaw technique, with its ligation of the splenic vessels while maintaining collateral perfusion from the gastroepiploic and short gastric vessels, provides a viable alternative in these situations [1]. The following case series highlights the importance of this approach.

## Case presentation

Case 1

**Figure 1:**
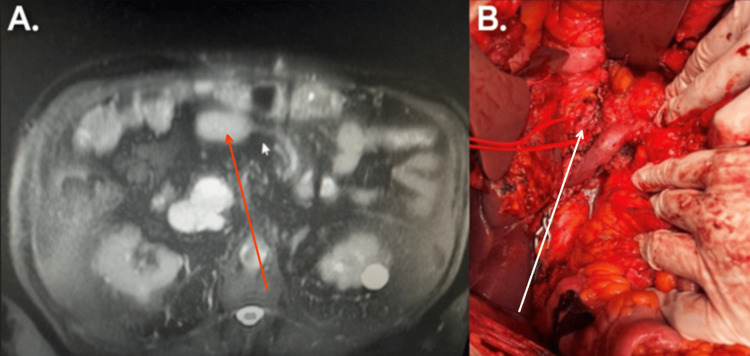
(A) CT scan showing a mucinous pancreatic neoplasm in the head of the pancreas (red arrow). (B) Intraoperative view of total pancreatectomy with right hemicolectomy (white arrow).

A 55-year-old female presented with a 5 cm mucinous pancreatic neoplasm invading the middle colic artery (Figure 1A). Preoperative imaging revealed prominent collateral veins and an accessory right hepatic artery arising from the superior mesenteric artery. The patient underwent spleen-preserving total pancreatectomy and extended right colectomy using the Warshaw technique (Figure 1B). Proximal splenic vessels were ligated at their origin, and the spleen’s blood supply was maintained through collateral vessels from the hilum, including the short gastric and left gastroepiploic vessels. Postoperatively, a CT scan revealed infarction of the upper pole, which resolved within two weeks.

Case 2

**Figure 2:**
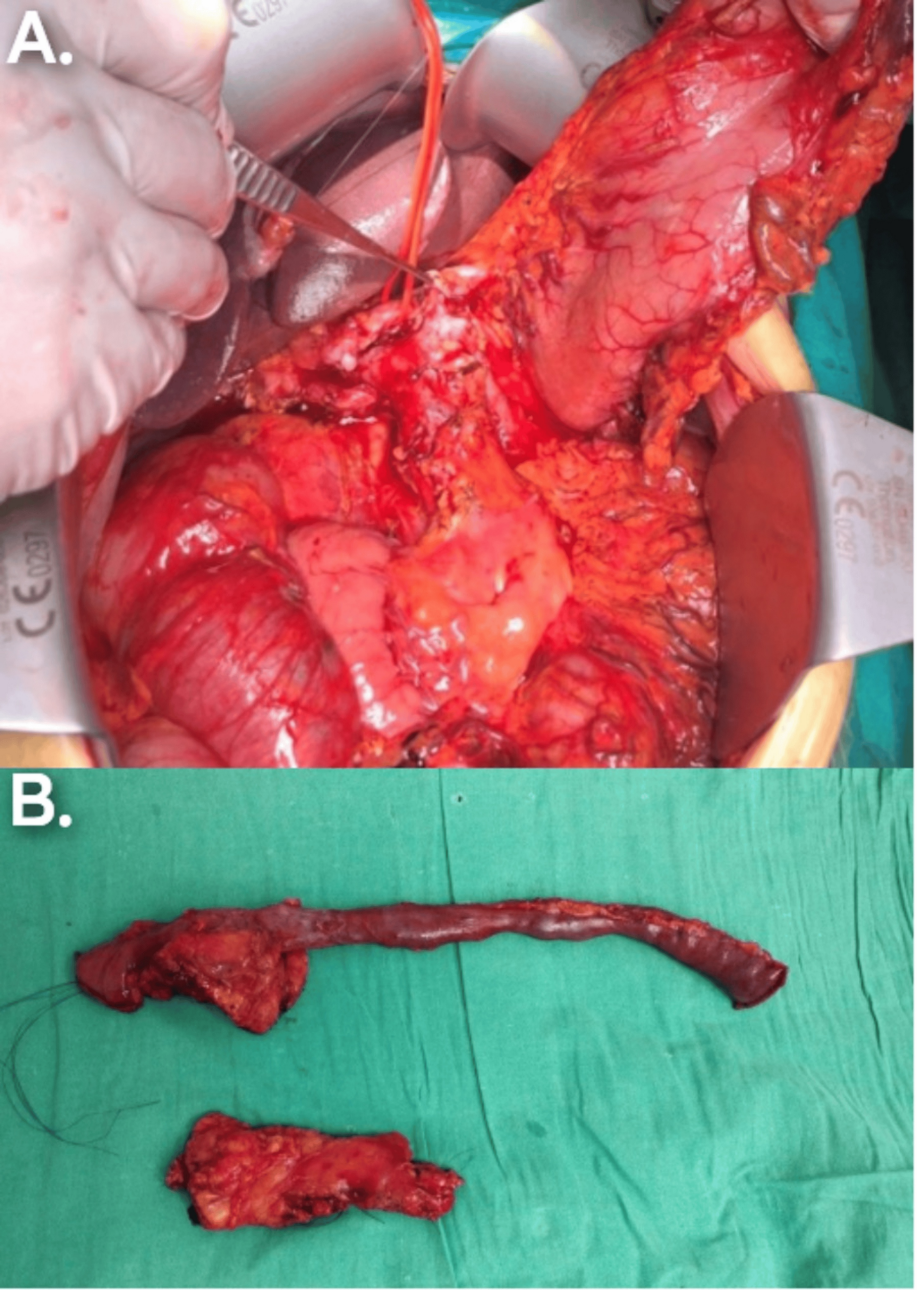
(A, B) Total pancreatectomy performed in a patient with high BMI and a soft pancreas, where dissection of the splenic vessels was challenging due to their intraparenchymal course.

**Figure 3:**
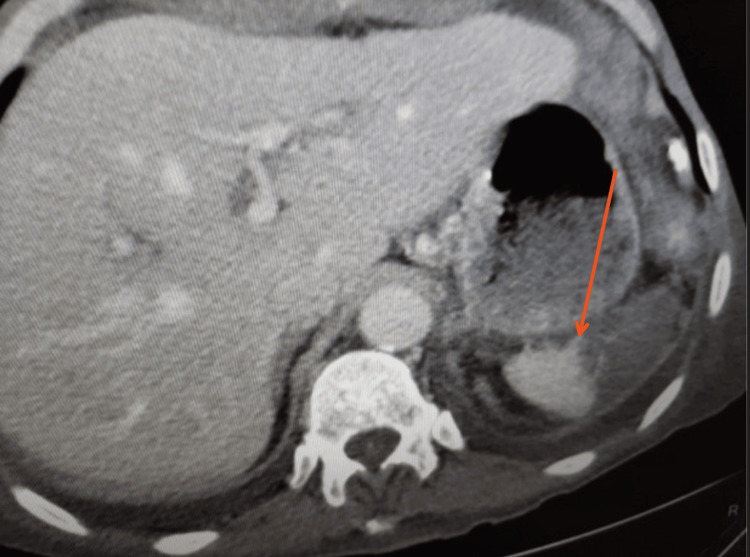
CT scan showing a splenic infarct (red arrow) following the Warshaw procedure, which resolved three months post-surgery.

A 65-year-old male with adenocarcinoma of the pancreatic head and a BMI of 40 underwent total pancreatectomy due to a high Fistula Risk Score. In addition to the high BMI, the soft pancreas and 2 mm pancreatic duct made the pancreaticojejunostomy (PJ) technically challenging, with a high incidence of POPF. Although a Kimura procedure was initially planned, the fatty infiltration of the peripancreatic tissue and the difficulty in fully dissecting the splenic vessels made the Warshaw procedure a feasible and effective alternative (Figure 2A, 2B). A splenic infarct was documented, which resolved without further sequelae three months later (Figure 3).

Case 3

**Figure 4:**
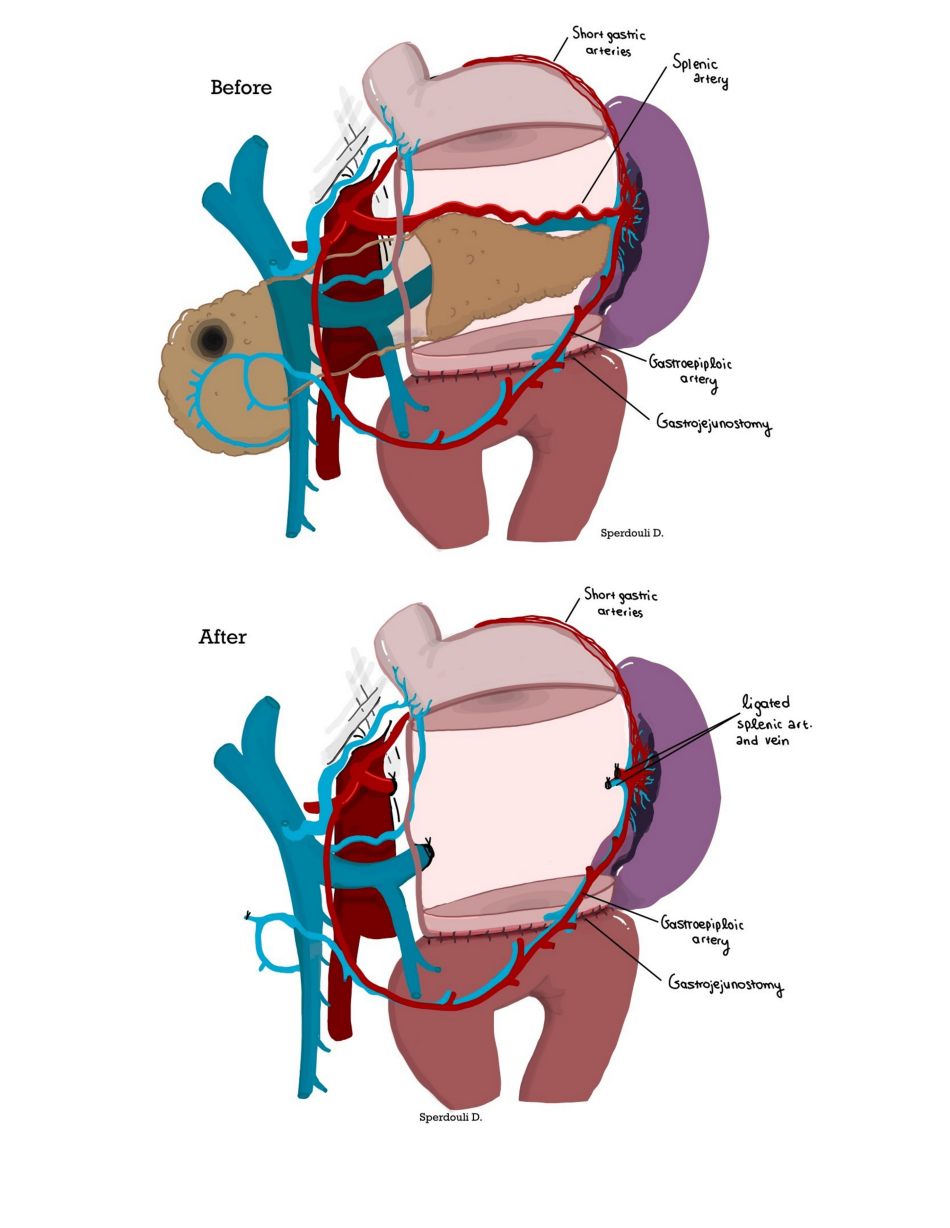
Warshaw procedure in a patient with a history of gastrectomy and GJ, demonstrating proximal splenic vessel ligation with preservation of the short gastric veins and left gastroepiploic artery and vein In this case, the spleen’s blood supply is maintained through collateral vessels to the splenic hilum. The patient had an uneventful postoperative recovery. GJ, gastrojejunostomy Image credit: Despoina Sperdouli

A 50-year-old male presented with a 5 cm serous cystadenoma at the head of the pancreas, accompanied by weight loss and severe discomfort due to gastric compression. The patient had previously undergone antrectomy and Billroth II reconstruction for a gastric ulcer 20 years ago. He underwent spleen-preserving total pancreatectomy. Due to the soft texture of the pancreas and the small pancreatic ducts, PJ reconstruction posed a high risk for POPF. In such cases, completion of distal pancreatectomy, with or without spleen preservation, is considered safe. In this case, the gastrojejunostomy (GJ) was intramesocolic, which complicated the dissection of the pancreas and associated vessels. The Warshaw procedure was performed using a stapling device at the tail of the pancreas, close to the splenic hilum (Figure 4).

## Discussion

The presentation of this technique is of significant interest due to the advantages it offers compared to the traditional splenectomy approach. However, it is also important to acknowledge the potential limitations of the technique.

In his 2009 review, Warshaw included multiple studies demonstrating the superiority of the Warshaw technique [5]. Specifically, patients undergoing spleen-preserving operations had fewer intraoperative complications, lower rates of postoperative infections, shorter operative times, and reduced hospital stays [5]. In the long term, these patients are less affected by complications associated with splenectomy, although issues related to spleen perfusion and the development of gastric varices may arise [5].

Another valuable comparison is between spleen-preserving techniques, such as the Warshaw technique, and non-spleen-preserving methods, particularly the Kimura technique. Fernández-Cruz et al. reported that the Warshaw technique was associated with shorter operative times and reduced blood loss [9]. In addition, Warshaw noted that the Warshaw technique was linked to splenic ischemic sequelae, whereas spleen-preserving methods were associated with thrombotic and bleeding complications involving the splenic vessels [5]. Two cases in this study exhibited transient infarction of the upper pole, which resolved spontaneously. Other studies, such as that of Paiella et al., found no significant differences in postoperative complication rates between the two techniques [6]. It is also important to note that spleen vessel-preserving techniques are generally more technically demanding, particularly in cases where the splenic vessels are intraparenchymal [7].

One of the main advantages of the Warshaw technique over distal pancreatectomy with splenectomy is spleen preservation. Patients who undergo splenectomy or those who are functionally asplenic face an increased risk of infections from encapsulated bacteria and require immunization against pneumococcus, meningococcus, and hemophilus [10]. Splenectomy also raises the risk of thrombotic incidents involving the portal, mesenteric, and splenic veins, as well as pulmonary embolism, due to increased prothrombotic factors postoperatively [10]. Stamou et al., in a prospective study, showed that the incidence of extrahepatic portal system thrombosis was as high as 5% in splenectomy patients [11]. Additionally, functional asplenia is associated with a higher risk of both hematological and solid cancers, potentially due to the loss of anti-tumor immunity [10,12].

In our experience, the Warshaw technique is crucial and offers a reliable alternative in cases involving high BMI, soft pancreas, and the challenging preservation of splenic vessels. Furthermore, if the tumor invades the coronary vein or inferior mesenteric vein and requires sacrifice, the Warshaw technique is advantageous in avoiding left portal hypertension and gastric congestion. This is particularly important in patients with a history of gastrectomy, as the technique preserves the short gastric veins and minimizes gastric congestion. Technically, it is less demanding when using a stapler device and transecting the pancreatic tail near the splenic hilum, without the need to disturb the GJ anastomosis. This manuscript aims to emphasize the value of the Warshaw technique in technically demanding distal or total pancreatectomies, where spleen preservation remains the primary objective.

## Conclusions

Despite significant progress in managing pancreatic cancer, it remains one of the deadliest cancers. Any modifications to current treatments, particularly surgical approaches, that offer favorable outcomes for patients are worth considering. This case series aims to highlight the Warshaw technique as such an innovation. It presents a valuable approach in spleen-preserving total pancreatectomy, with favorable outcomes in preserving splenic blood supply and reducing postoperative complications. Its versatility makes it applicable in a range of clinical scenarios, further reinforcing its importance in pancreatic surgery.
